# Case Report: Neuro-ophthalmic manifestations of petroclival meningioma

**DOI:** 10.3389/fopht.2026.1841667

**Published:** 2026-05-21

**Authors:** Natalie Lanners, Ritu Sampige, Safa Ibrahim, Dina Abdelsalam, Andrew G. Lee

**Affiliations:** 1 McGovern Medical School, University of Texas Health Science Center at Houston, Houston, TX, United States; 2 School of Medicine, Baylor College of Medicine, Houston, TX, United States; 3 Department of Ophthalmology, Blanton Eye Institute, Houston Methodist Hospital, Houston, TX, United States; 4 Departments of Ophthalmology, Neurology, and Neurosurgery, Weill Cornell Medicine, New York, NY, United States; 5 Department of Ophthalmology, Cullen Eye Institute, Baylor College of Medicine, Houston, TX, United States; 6 Department of Ophthalmology, University of Texas MD Anderson Cancer Center, Houston, TX, United States; 7 Texas A&M College of Medicine, Bryan, TX, United States; 8 Department of Ophthalmology, University of Iowa Hospitals and Clinics, Iowa City, IA, United States

**Keywords:** abducens nerve palsy, cranial nerve palsies, dysphagia, iatrogenic complication, petroclival meningioma

## Abstract

**Purpose:**

To describe the neuro-ophthalmic manifestations of petroclival meningioma including multiple cranial neuropathies (e.g., trigeminal (V), abducens (VI), facial (VII), vestibulocochlear (VIII), glossopharyngeal (IX), vagus (X), accessory (XI), and hypoglossal (XII) dysfunction).

**Background:**

Petroclival meningiomas (PCM) are uncommon, benign skull base tumors that often present gradually but can lead to significant morbidity and mortality due to brainstem and lower cranial nerve involvement.

**Case presentation:**

A 42-year-old woman presented with gradual and progressive neck pain, headaches, and dysphagia. Neuroimaging showed a large petroclival meningioma with secondary obstructive hydrocephalus. She underwent staged surgical resections with near-gross total tumor resection. The patient subsequently developed new cranial nerve VI and VIII deficits. We also include a patient perspective of her condition as a unique aspect of the evaluation of PCM.

**Conclusion:**

PCM can present with multiple progressive cranial neuropathies due to tumor effects or as a consequence of surgical or radiation therapy. We believe that including a patient perspective in clinical reports might improve physician empathy and understanding of the patient’s condition and course beyond the traditional evaluation and management paradigms for brain tumors including PCM.

## Introduction

Petroclival meningiomas (PCM) are uncommon, benign tumors arising at the junction of the clivus and petrous bone, frequently abutting the brainstem and multiple cranial nerves (CN). The deep location and indolent growth pattern of PCM can lead to delayed diagnosis (1). CN dysfunction in PCM may involve the trigeminal, abducens, facial, vestibulocochlear, glossopharyngeal, vagus, accessory, or hypoglossal CN. Post treatment CN deficits are also common ranging between 20% to 50%, depending on tumor size, extent of resection, and surgical approach (2–5). The risk is highest with more aggressive resections and complex skull base approaches, and deficits may be transient or permanent (6). We describe the neuro-ophthalmic manifestations of PCM and review the literature.

## Case report

A 42-year-old female presented with insidious onset of neck pain, headache, and progressive dysphagia. Her past medical history included asthma. Her medications included albuterol and tiotropium. The remainder of her past medical, ocular, surgical, family, social, and allergic histories were non-contributory.

In November 2024, she consulted a sports medicine physician for worsening neck pain and tenderness. Cervical spine radiographs showed mild degenerative changes at C7–T1 with otherwise normal alignment. She was prescribed steroids, muscle relaxants, and physical therapy.

In December 2024, she presented to urgent care with acute hypoxemia (oxygen saturation in the 70% range) and was admitted to the intensive care unit (ICU). Respiratory workup identified concurrent influenza with superimposed streptococcal pneumonia. Despite treatment, she progressed to respiratory failure requiring intubation and tracheostomy. The patient’s acute presentation and rapid deterioration prompted neuroimaging.

**Figure 1:**
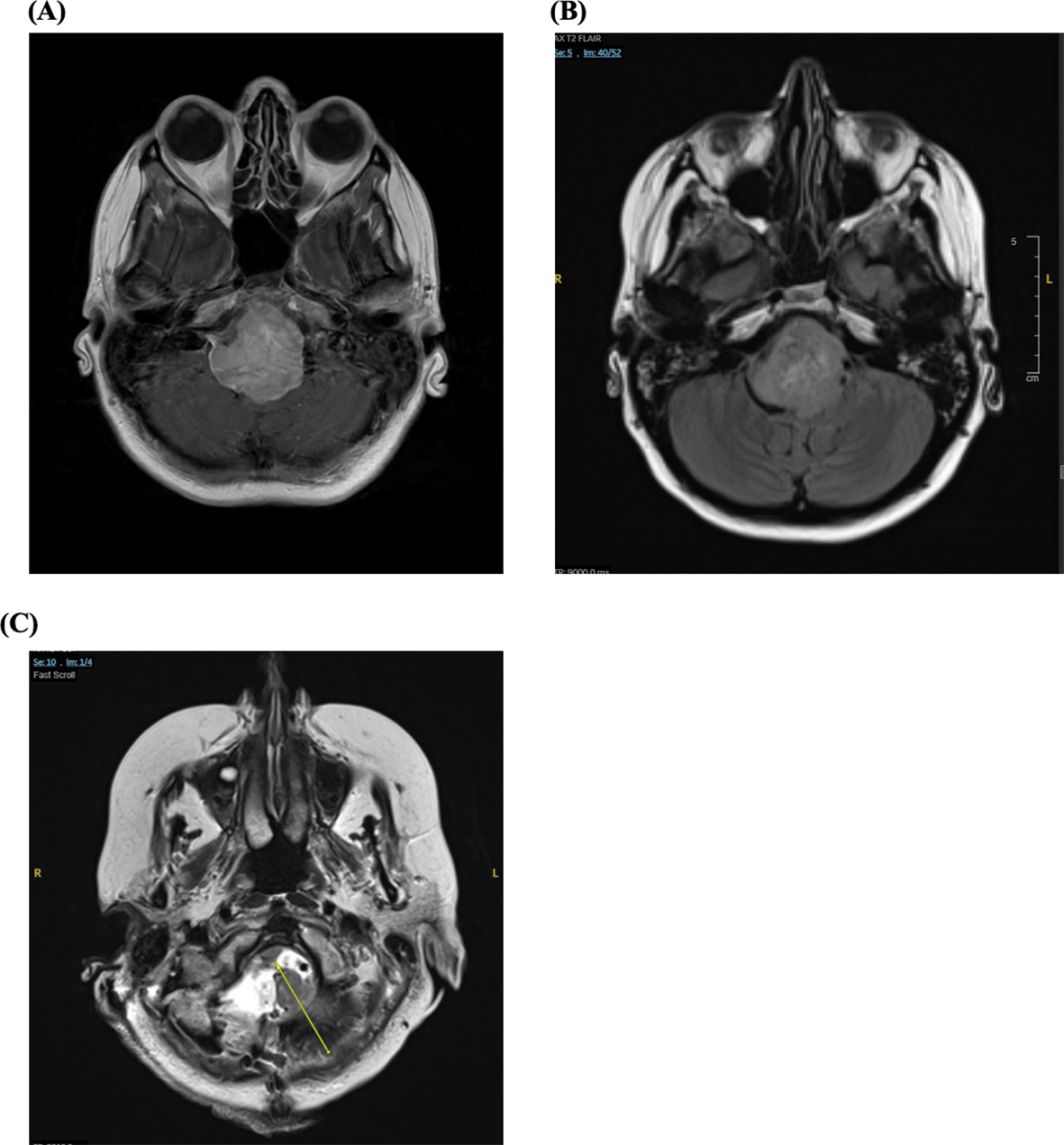
Axial magnetic resonance images (MRI) demonstrating a large right petroclival meningioma obstructing cerebrospinal fluid (CSF) flow through the fourth ventricle. **(A)** T1-weighted post-contrast image shows the avidly enhancing petroclival mass. **(B)** The T2 FLAIR sequence illustrates secondary hydrocephalus due to CSF outflow obstruction. **(C)** Postoperative axial T2-weighted MRI demonstrating near-total resection of the right petroclival mass, with only a sub-centimeter focus of residual enhancement at the right anterior foramen magnum margin, indicating minimal residual tumor (*yellow arrow*).

A magnetic resonance imaging (MRI) was obtained, revealing a large right-sided petroclival mass causing brainstem compression and obstructive hydrocephalus (**Figure 1**). She was subsequently transferred to Houston Methodist Hospital for neurosurgical evaluation.

In January 2025, she underwent an urgent ventriculoperitoneal shunt placement for obstructive hydrocephalus and a percutaneous endoscopic gastrostomy (PEG) tube for worsening dysphagia. Both deficits were present preoperatively: dysphagia and hoarseness were attributable to tumor mass effect on CN IX and X, while elevated intracranial pressure reflected CSF outflow obstruction. In February 2025, she underwent the first stage of surgical resection; pathology confirmed a World Health Organization (WHO) grade I meningioma. Shortly thereafter, she developed an acute-onset, painless horizontal binocular diplopia. Inpatient ophthalmology evaluation revealed a new right CN VI palsy — absent preoperatively and therefore representing a postoperative iatrogenic deficit — which was initially managed with patching. She continued to experience persistent dysphagia and hoarseness following surgery, consistent with preoperative CN IX and X dysfunction that did not recover after the first resection. In late March 2025, she underwent the second stage of surgical resection to excise the residual tumor. Postoperative MRI showed near-total resection, with only a small residual focus (<1 cm) at the foramen magnum margin (**Figure 1**). In early April 2025, she noted new right-sided sensorineural hearing loss consistent with CN VIII involvement, likely attributable to surgical insult during the second resection and representing an additional new postoperative deficit. Once stabilized, she was discharged home but was readmitted in early July 2025 due to functional decline and inability to perform basic activities of daily living, requiring transfer to inpatient rehabilitation.

**Figure 2:**
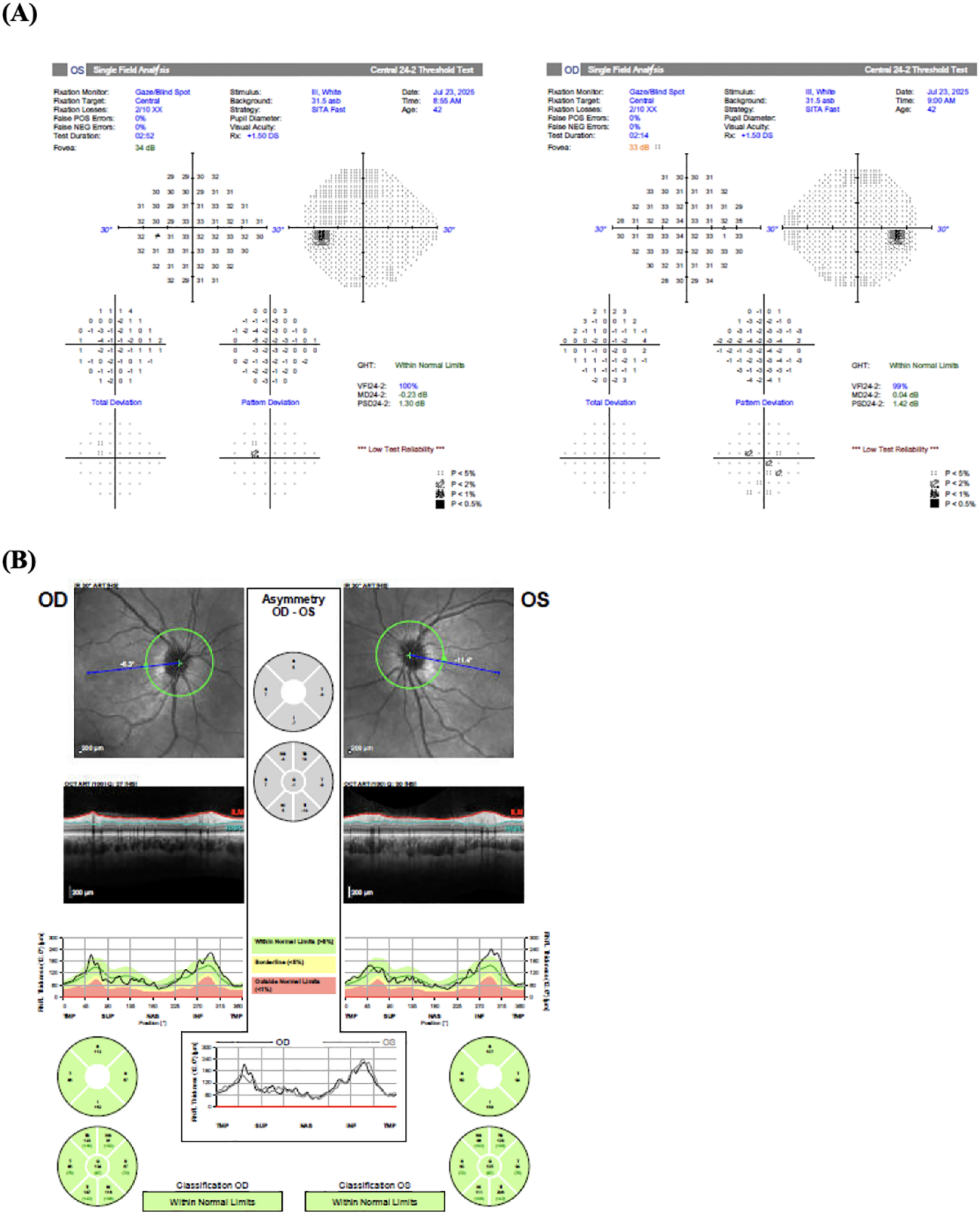
**(A)** Humphrey visual field testing demonstrated minimal deviations. **(B)** Optical coherence tomography showed no evidence of papilledema.

In July 2025, the patient was evaluated by neuro-ophthalmology for her persistent diplopia. On examination, visual acuities were 20/25 in the right eye (OD) and 20/20 in the left eye (OS). Intraocular pressures were within normal limits. Color vision was intact bilaterally. Humphrey visual fields showed minimal deviations, and optical coherence tomography revealed no papilledema (**Figure 2**). Pupillary examination, including relative afferent pupillary defect (RAPD), was normal with no anisocoria — arguing against CN III involvement and confirming intact CN II function. Extraocular motility testing revealed a right esotropia of 50 prism diopters with a −4 abduction deficit in the right eye consistent with a right CN VI palsy; the left eye moved normally. There was no ptosis or proptosis. Pertinent negatives on clinical assessment included intact facial sensation (CN V) and facial symmetry (CN VII). Routine laboratory evaluation – including vitamin B12, folate, methylmalonic acid, homocysteine, thyroid function panel, erythrocyte sedimentation rate, C-reactive protein, myasthenia gravis antibody panel, syphilis serology, Borrelia burgdorferi (Lyme disease) serology, Bartonella antibody panel, and tuberculosis testing – was unremarkable. The patient’s computed tomography scan showed no evidence of tumor recurrence or hydrocephalus. Therefore, the clinical findings were consistent with a surgically induced right CN VI palsy. **Table 1** summarizes the patient’s clinical course and key interventions chronologically.

**Table 1:** Timeline of clinical course.

Date	Event/Findings	Diagnostics	Management/Outcomes
Mid-2024	Gradual onset of neck pain, headache, and progressive dysphagia	—	Managed conservatively
Nov 2024	Worsening neck pain; sports medicine evaluation	Cervical spine X-rays: mild C7–T1 degenerative changes	Treated with steroids, Flexeril, and physical therapy
Early Dec 2024	Presented to urgent care with acute hypoxemia (SpO_2_ 70s) ➔ ICU admission	Positive for influenza; chest X-ray: streptococcal pneumonia	Antibiotics, intubation, tracheostomy due to respiratory failure
Late Dec 2024	Rapid clinical deterioration	MRI brain: large right petroclival mass compressing brainstem with obstructive hydrocephalus	Transferred for neurosurgical evaluation
Jan 2025	Neurosurgical intervention	—	Ventriculoperitoneal shunt placed; PEG tube inserted (preoperative dysphagia from CN IX-X)
Feb 2025	Stage 1 meningioma resection	Pathology: WHO grade I meningioma	New postoperative right CN VI palsy (iatrogenic) → horizontal diplopia; managed with patching
Mar 2025	Stage 2 meningioma resection	Post-op MRI: near-total resection with < 1 cm residual at foramen magnum	Persistent dysphagia and hoarseness (preoperative CN IX-X); CN VI palsy persistent
Apr 2025	Right-sided hearing loss	Audiologic testing: sensorineural hearing loss (CN VIII, new postoperative deficit)	Managed conservatively
Early Jul 2025	Readmitted for functional decline → inpatient rehabilitation	Routine labs and CT: no tumor recurrence	Initiated multidisciplinary rehabilitation
Late Jul 2025	Evaluation of persistent diplopia	BCVA 20/25 OD, 20/20 OS; pupils equal, no RAPD; color vision intact; HVF: minimal deviations; OCT: no papilledema; EOM: 50 PD right esotropia, −4 right abduction deficit;	Diagnosis: surgically induced right CN VI palsy; manage with vision therapy
Nov 2025	Surveillance imaging	MRI brain: no residual or recurrent tumor	Ongoing rehabilitation; planned swallow reassessment
Feb 2026	Strabismus surgery for persistent diplopia	Clinical improvement post-procedure	Notable improvement in diplopia; Persistent but improving VIII, IX, X deficits; no frequent suctioning required though PEG-dependent

BCVA, best-corrected visual acuity; CN, cranial nerve; CT, computed tomography; EOM, extraocular movements; ICU, intensive care unit; MRI, magnetic resonance imaging; OD, right eye; OS, left eye; PD, prism diopters; PEG, percutaneous endoscopic gastrostomy; RAPD, relative afferent pupillary defect; WHO, World Health Organization.

## Follow-up and outcomes

At the time of this report, the patient is actively participating in comprehensive rehabilitation, including physical therapy, occupational therapy, speech therapy, and vision therapy, with good adherence and tolerability. Over the past year, she improved but continued to experience persistent deficits, including CN IX and X dysfunction, as evidenced by ongoing PEG dependence, CN VI-associated diplopia, and CN VIII-associated right-sided hearing loss. Her most recent surveillance MRI, performed in November 2025, demonstrated no evidence of residual or recurrent tumor.

The patient’s ocular prognosis was initially uncertain, as recovery of abducens nerve function is variable and depends on factors such as nerve preservation and extent of resection. Recovery is generally more favorable in cases of subtotal resection, where the nerve is more likely to remain anatomically intact. Given that this patient underwent near-total resection, full recovery was less certain. Due to persistent diplopia requiring daily patching, she underwent strabismus surgery in February 2026, resulting in notable improvement.

Neurologically, the presence of persistent CN IX-X deficits both before and after surgery suggests a more chronic, likely irreversible brainstem injury. Her swallowing function has greatly improved with speech therapy. As of February 2026, she has not required frequent suctioning, has had fewer choking episodes, and reports decreased nasal regurgitation during swallowing. A repeat swallowing evaluation is planned to assess readiness for PEG tube removal. Overall, the patient remains clinically stable, with preserved visual acuity and no evidence of tumor recurrence, though long-term supportive care will likely be required.

## Discussion

PCM arises at the junction of the clivus and petrous bone, placing the tumor in intimate contact with the brainstem and multiple cranial nerves traversing the posterior fossa (1). The anatomical basis for the CN deficits seen in this case is directly related to this location: the abducens nerve (CN VI) has an unusually long intracranial course and passes through Dorello’s canal near the petrous apex, making it vulnerable to both mass effect and surgical traction; the vestibulocochlear nerve (CN VIII) travels in the internal auditory canal in close proximity to the tumor; and the lower CNs (IX–XII) exit through the jugular foramen and hypoglossal canal at the skull base, where tumor compression produces the dysphagia, hoarseness, and aspiration seen preoperatively in this patient (1, 2). The trigeminal nerve (CN V) courses along the petroclival ridge and is frequently compressed, accounting for its high prevalence in published series (7). Understanding these anatomical relationships is essential for anticipating which deficits may be preoperative versus postoperative in origin, and for counseling patients regarding surgical risk (5).

PCM as well as the treatment of PCM can result in multiple CN palsies including ocular motor CN palsy. Preoperative CN dysfunction was reported in the majority of patients with PCM (60–75%) with CN V and CN VIII most frequently affected, followed by CN II, CN III and VI, and lower CNs (CN IX-XI) in invasive tumors (3, 4, 7–9). New postoperative CN deficits occurred in up to 40–44% of patients across contemporary cohorts, though most were transient and improved within months of follow-up (4, 9). **Table 2** summarizes the literature on the ocular motor CN palsies in PCM.

**Table 2:** Summary of pertinent prior studies reporting cranial nerve (CN) deficits before and after petroclival meningioma resections.

Study	N (patients)	Preoperative CN deficits	Most affected nerves	New postoperative CN deficits	Recovery/Persistence	Notes
Wagner et al., 2022 (*Cancers*) (9)	64	73.4%	V, VIII, II	~44% (mostly transient)	Persistent if preoperative	Supports tailored STR
Schackert et al., 2022 (*J Neuro-Oncol*) (4)	158	Majority	V, VIII; lower CNs in invasive tumors	Up to 40%	Lower CNs rarely recovered	Standard craniotomy approaches
Nanda et al., 2011 (*J Neurosurg*) (7)	114	Majority	V, VIII	44%	Many transient; persistence linked to pre-op palsy	STR vs GTR showed no functional advantage
Bernard et al., 2019 (*Neurochirurgie*) (10)	154	High rate	V, VI	Frequent, esp. V & VI	Lower CN deficits are more permanent	Multicenter, long-term cohort
Di Carlo et al., 2020 (*Acta Neurochir*, meta-analysis) (5)	1,200+ pooled	60–70% pooled	V, VIII	Pooled new CN deficit ~39%	Persistent if pre-op severe	Compared surgical approaches
Khaleghi et al., 2025 (*J Clin Neurosci*, meta-analysis) (12)	2,884 pooled	Reported facial nerve deficit rates	VII	Higher in petroclival vs posterior petrous	Persistent in subset	Large comparative meta-analysis

Across historical and contemporary cohorts, preoperative CN deficits were highly prevalent (60–75%), most often affecting the trigeminal (CN V) and vestibulocochlear (CN VIII) nerves, followed by optic (CN II), oculomotor/abducens (CN III/VI), and lower CNs (CN IX–XI) in invasive tumors. New postoperative CN deficits occurred in up to 44% of cases, though most were transient and improved with follow-up. Persistent dysfunction was closely associated with preoperative palsy, particularly in lower CNs. Systematic reviews and meta-analyses reinforced that the surgical approach influences the risk profile, while subtotal resection (STR) may minimize morbidity without increasing recurrence (5, 12). CN, cranial nerve; GTR, gross total resection; STR, subtotal resection.

Persistent deficits were strongly predicted by preexisting palsy – particularly in lower CNs, which are less likely to recover and may cause airway or swallowing dysfunction (7, 10, 11). Therefore, patients with pre-existing deficits are less likely to experience full recovery after surgery, and the risk of permanent neurological morbidity is increased in this group. Meta-analyses and multicenter studies continue to highlight that CN V and VI are most vulnerable to new postoperative injury, regardless of approach (5). While aggressive gross-total resection (GTR) can achieve excellent radiographic control, several series advocate for function-preserving subtotal resection (STR) to reduce morbidity without compromising long-term outcomes (9, 12). Therefore, selective subtotal resection may be favored in patients with extensive preoperative CN involvement to minimize additional morbidity, as gross total resection is associated with a higher risk of new deficits without a significant reduction in recurrence rates (3, 12). Careful preoperative assessment and individualized surgical planning are essential to optimize functional preservation. PCM can present with lower CN deficits which pose a greater risk for morbidity (dysphagia) and mortality risk (aspiration) (2, 12).

## Patient perspective

“I used to be very active and healthy. I ran several times a week and even completed half marathons. About three years ago, I started having persistent neck pain that gradually got worse and eventually limited my ability to run. I also developed headaches and fatigue, which I initially brushed off as being a busy mom of three. As things worsened, I sought care, but answers weren’t clear at first. I was shocked when I was told my oxygen levels were so low in that urgent care. I began having trouble walking and breathing, and my husband knew something was seriously wrong and pushed me to go to the hospital. I became very sick quickly and don’t remember much after the first few days, especially because I was intubated. My family had to strongly advocate for me as my condition declined. Eventually, imaging revealed a tumor, and I was transferred for specialized care. After my resection surgeries, recovery was long and challenging. I experienced significant weakness and had to work hard in therapy to regain my strength and independence. Communication was especially frustrating early on. Today, I’ve made meaningful progress. I’m still working on swallowing and strength, but I’m improving every day. I truly believe that staying positive, along with the support of my family and care team, played a huge role in helping me recover”.

The patient perspective above provides valuable insight into the lived experience of PCM, including the diagnostic odyssey, the acute respiratory decompensation that ultimately triggered diagnosis, the prolonged surgical and rehabilitation course, and the significant psychosocial impact on a young, active patient. However, we acknowledge that this represents a single patient’s experience and cannot be considered broadly representative. Future studies incorporating validated questionnaire-based patient-reported outcome measures across larger PCM cohorts are needed to draw generalizable conclusions about patient experience.

## Conclusion

PCM can present insidiously with vague, non-specific symptoms including headache and neck pain that may be misattributed to benign musculoskeletal or infectious etiologies, causing diagnostic delays. Clinicians should maintain a high index of suspicion for skull base pathology in patients with lower CN neuropathy (dysphagia, hoarseness, or aspiration), as these findings may be the first and most prominent manifestation of PCM. In this case, progressive lower CN dysfunction ultimately triggered the neuroimaging that revealed the tumor. The key anatomical teaching point is that CN VI is particularly vulnerable at the petroclival junction due to its long intracranial course through Dorello’s canal; new postoperative abducens palsy should therefore be anticipated, patients should be informed of this risk preoperatively, and baseline neuro-ophthalmic documentation of pertinent positive and negative findings is essential before surgery. The staged temporal pattern of deficits observed here — preoperative CN IX/X dysfunction followed by postoperative CN VI palsy after the first resection, and new CN VIII deficit after the second — illustrates how morbidity can accumulate across multiple interventions and underscores the importance of shared decision-making when planning staged resections in patients with existing CN deficits.

## Data Availability

The original contributions presented in the study are included in the article/supplementary material. Further inquiries can be directed to the corresponding author.
